# An interesting clinical case. New therapies in Dissociative Identity Disorder.

**DOI:** 10.1192/j.eurpsy.2023.2064

**Published:** 2023-07-19

**Authors:** P. García Vázquez, E. Seijo Zazo, C. Vilellla Martin, A. Serrano García, C. M. Franch Pato, E. Martína Gil, C. Alvarez Vazquez

**Affiliations:** 1 HUCA, Oviedo; 2 CAULE, LEON, Spain

## Abstract

**Introduction:**

Dissociative identity disorder (DID) also referred as multiple personality disorder is a chronic post-traumatic condition. It is characterized according to DSM-5 by “disruption of identity characterized by two or more distinct personality states”, with “marked discontinuity in sense of self… accompanied by related alterations in affect, behavior, consciousness, memory, perception, cognition, and/or sensory-motor functioning.”

**Objectives:**

Here, we present a case of a 33-year-old Caucasian female with no psychiatric history until 2 years ago, privately. The patient is admitted to the Psychiatry Service due to worsening. During admission, consultations are made to the Neurology Service and the Neurophysiology Service, who request an electroencephalogram, an MRI and a brain scan, resulting in normality.

After discharge, she returns home with her parents, and the crisis become more frequent and of longer duration. She acknowledges that during these periods she is dominated by her alternate personality, which she is unaware of until her family informs her. This personality is a demon, who verbally assaults and even physically threatens her surroundings, and can hardly be controlled by the prayers of her family.

**Methods:**

Despite psychopharmacological treatment, as well as the cognitive-behavioral therapy carried out by the patient for more than two years, there was no improvement. Once she comes to the consultation, it is decided to carry out a therapy guided by the central Rogerian attitudes, originating a process of empathic resonance of the therapist, which influences the experience of the patient. Three main interventions are carried out, the awareness of the disease, the regulation of the intensity of this experience, to maintain the attention and the exploration of what guides the change. After carrying out this intervention, the patient is currently asymptomatic.

**Results:**

Currently, there are not evidence-based treatment guidelines. The most common approach is individual psychodynamic psychotherapy according to practice-based guidelines initiated by the International Society for the Study of Trauma and Dissociation.

To handle the present case, we used a model with two pillars, the patient’s commitment and the investigation of microprocesses within a process of experiential exploration, in which the therapist is a facilitator of reflective attention and experimental awareness.

**Conclusions:**

The torpid evolution suffered by the patient, with little clinical improvement to the interventions carried out, and the absence of evidence on the treatment, led to a therapeutic approach focused on the empathic resonance process of the therapist, with good results.

**Disclosure of Interest:**

None Declared

